# Single-Atom Catalysts for Hydrogen Evolution Reaction: The Role of Supports, Coordination Environments, and Synergistic Effects

**DOI:** 10.3390/nano15151175

**Published:** 2025-07-30

**Authors:** Zhuoying Liang, Yu Zhang, Linli Liu, Miaolun Jiao, Chenliang Ye

**Affiliations:** 1 Hebei Key Laboratory of Energy Storage Technology and Integrated Energy Utilization, North China Electric Power University, Baoding 071003, China; 2 Department of Chemistry, Tsinghua University, Beijing 100084, China; 3 Testing Center of the Geophysical Exploration Academy of China Metallurgical Bureau, Baoding 071051, China

**Keywords:** hydrogen evolution reaction, single-atom catalysts, support, coordination environment, synergistic effects

## Abstract

Single-atom catalysts (SACs) have emerged as highly promising catalytic materials for the hydrogen evolution reaction (HER), attributed to their maximal atomic utilization efficiency and unique electronic configurations. Many structure parameters can influence the catalytic performance of SACs for HER, and the intrinsic advantages of SACs for HER still need to be summarized. This review systematically summarizes recent advances in SACs for HER. It discusses various types of SACs (including those based on Pt, Co, Ru, Ni, Cu, and other metals) applied in HER, and elaborates the critical factors influencing catalytic performance—specifically, the supports, coordination environments, and synergistic effects of these SACs. Furthermore, current research challenges and future perspectives in this rapidly developing field are also outlined.

## 1. Introduction

The energy crisis and environmental pollution are great challenges for human beings. As a renewable energy, hydrogen holds significant potential for next-generation clean energy [1,2,3,4]. In earlier decades, hydrogen was mainly produced from the conversion of fossil fuel, which has caused severe environmental pollution. At present, with the development of electrochemistry technology, water splitting is viewed as a promising process for generating green hydrogen, and has attracted great attention for academic research and the chemical industry [5,6,7,8,9]. During the water splitting process, the oxygen evolution reaction (OER) occurs on the anode and HER occurs on the cathode. As one of the important electrode reactions of water splitting, the HER process plays a key role in producing hydrogen.

HER encompasses three key steps: the Volmer, Heyrovsky, and Tafel reactions [10]. The Volmer reaction involves hydrogen adsorption, where reactants (H^+^ or H_2_O) adsorb onto the catalyst surface and accept electrons, forming adsorbed hydrogen atoms (H*). The subsequent Heyrovsky reaction, an electrochemical desorption process, entails the combination of adsorbed hydrogen (H*) with H^+^ (or H_2_O) in the solution, accompanied by electron uptake to generate H_2_. The Tafel reaction, a chemical desorption step, is characterized by the direct coupling of two adsorbed hydrogen atoms (H*) to yield H_2_.

Advanced HER catalysts on the cathode play a key function to achieve high hydrogen production efficiency [11,12,13,14,15]. At present, Pt-based nanocatalysts are the most widely applied catalytic materials for HER, and exhibit moderate activity but face substantial limitations: (1) high cost due to reliance on precious metals; (2) a risk of dissolution and loss of atoms on the surface of nanoparticles in extreme electrochemical environments; and (3) low atom utilization, where only surface atoms participate in catalysis. These challenges underscore the urgent need for advanced catalysts that maximize metal utilization and improve the catalytic performance. In addition to Pt-based catalysts, non-noble metal nanocatalysts, such as Co, Ru, Ni, and Cu, have also been investigated for HER [16,17,18,19,20,21]. However, the catalytic activity and stability of these non-noble metal catalysts are still far from being suitable for industrial applications.

Zhang et al. first proposed the concept of SACs in 2011. They synthesized a highly active and stable Pt_1_/FeO_x_ catalyst for CO oxidation by a co-precipitation method [22]. Since then, SASCs have attracted great attention in catalysis, and numerous studies have focused on controlled synthesis, advanced characterization, and applications [23,24,25,26]. The fundamental distinction between SACs and traditional catalysts—such as nanoparticle and cluster catalysts—resides in the atomic-scale structure of their active sites and the associated electronic properties, and many interesting results show that SACs exhibit higher HER performance than that of traditional nanocatalysts [26,27,28,29,30]. The evolution of catalyst design has been accompanied by a continuous reduction in the size of the active phase; a decrease in particle dimensions inherently leads to an increase in the surface-to-volume ratio. Enabled by the rise in nanoscience and nanotechnology, this miniaturization process has progressed remarkably. SACs, featuring isolated metal atoms anchored on supports, offer transformative potential by addressing thesis limitations. SACs achieve near-100% atom utilization, drastically reducing metal loading, and have the potential to achieve high catalytic activity and stability due to their unique electronic structure. However, many factors (such as supports, coordination environments, and synergistic effects) can influence the catalytic performance of SACs for HER, and the intrinsic advantages of SACs for HER still need to be summarized.

Based on the great potential of SACs for HER, in this review, we systematically summarize the recent progress of SACs for HER. This review discusses extensive types of SACs (Pt, Co, Ru, Ni, Cu, and other metals) for HER, and carefully discloses the fundamental regulatory factors governing HER performance, including supports, coordination environments, and synergistic effects. We also summarize the current research challenges and future perspectives of SACs for HER. This review will be of great interest for designing high-efficiency HER catalysts.

## 2. Pt-Based Catalysts

Platinum (Pt) exhibits exceptional catalytic properties, characterized by near-zero overpotentials across extreme pH conditions and outstanding stability [27,28,29]. However, the prohibitive cost and scarcity of Pt significantly elevate catalyst expenses. Consequently, maximizing Pt atom utilization efficiency has emerged as a critical challenge in catalyst design. SACs address this challenge by dispersing active Pt atoms uniformly onto a support, effectively decoupling catalytic performance from particle size and shape constraints, thereby achieving near-100% Pt atom utilization [30,31]. Crucially, the HER performance of Pt SACs is governed by several key factors: the anchoring capability of the support material [32,33,34], the specific coordination environment of the isolated Pt atom [35,36], and synergistic effects arising between the single Pt atoms and adjacent metal atoms or nanoclusters [37,38].

### 2.1. Supports

Carbon-based materials are highly suitable supports for Pt SACs due to their inherent chemical stability, wide availability, and superior electrical conductivity [39,40]. However, the intrinsic limitation of unmodified carbon lies in its inherently weak binding affinity towards isolated metal atoms. This fundamental constraint leads to low Pt loading and poor dispersion on pristine carbon supports, rendering them inadequate for high-performance Pt SAC applications for HER.

To overcome this critical weakness, modifying the carbon support itself has emerged as a central strategy [41,42]. Introducing heteroatoms (e.g., N, S, P) creates structural defects and alters the electronic nature of the support surface, enhancing its ability to anchor single Pt atoms. For instance, Yang et al. employed a nitrogen-doped porous carbon framework (NDPCF) as the support to simultaneously confine Pt single atoms (Pt_SA_) and CoPt alloy nanocrystals (CoPt-Pt_SA_/NDPCF) [43]. The NDPCF not only provides an attachment site for the active components, but also prevents the erosion of Pt_SA_ and CoPt alloys by electrolytes, giving CoPt-Pt_SA_/NDPCF a long cycle durability. This engineered support enabled the catalyst to surpass commercial 10% Pt/C significantly in HER performance across acidic and alkaline conditions, possessing lower overpotentials, smaller Tafel slopes, higher current densities, higher turnover frequency (TOF), and exceptional durability. The outstanding synergy is primarily attributed to the co-support of Pt_SA_ and CoPt nanocrystals within the functionalized NDPCF scaffold.

**Figure 1:**
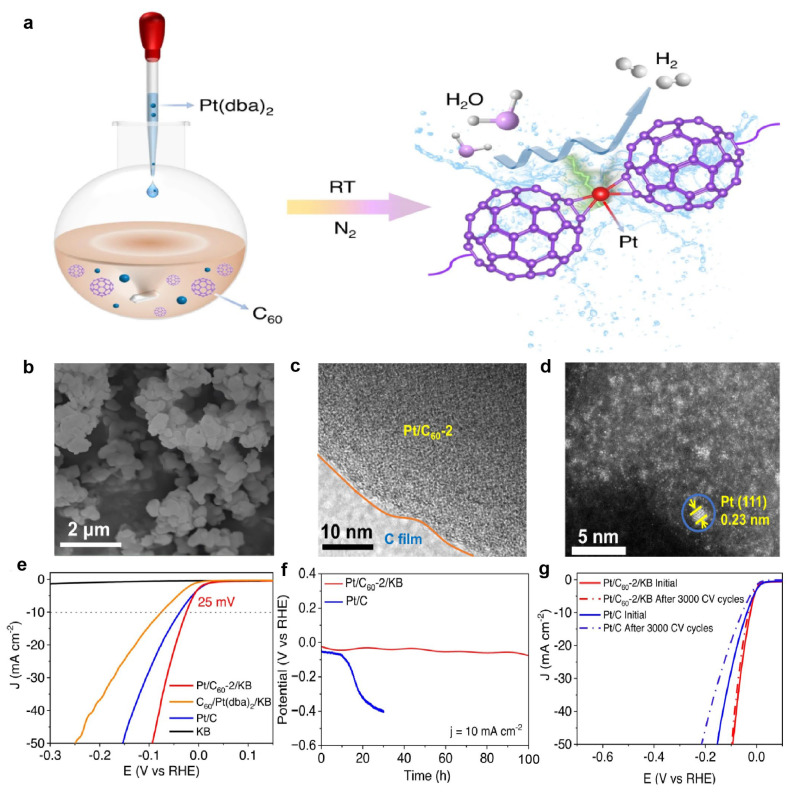
The synthetic scheme, morphological characteristics, and HER performance of Pt/C_60_-2 [44]. (**a**) Synthetic scheme of Pt/C_60_ catalysts; (**b**) SEM; (**c**) HRTEM; and (**d**) HAADF-STEM images of Pt/C_60_-2; (**e**) HER polarization curves for Pt/C_60_-2/KB, C_60_/Pt(dba)_2_/KB, Pt/C, and KB in 1 M KOH; (**f**) long-term stability test at a current density of 10 mA cm^−2^ in 1 M KOH; (**g**) LSV curves for Pt/C_60_-2/KB and Pt/C before and after 3000 catalytic cycles in 1 M KOH.

Beyond heteroatom-doped carbons, specific carbon allotropes offer unique structural properties conducive to ultra-high metal loading. Fullerene (C_60_) serves as an effective molecular support platform due to its high symmetry and distinct electron affinity, which facilitates direct interaction and immobilization of Pt atoms. Zhang et al. synthesized a Pt/C_60_-2 catalyst by replacing the dba ligands in Pt(dba)_2_ with C_60_ molecules (n(C_60_):n(Pt(dba)_2_) = 2:1). This direct integration onto the C_60_ support resulted in remarkably high Pt loading (21.54 wt%) (Figure 1a) [44]. Structural characterization (SEM, TEM, HAADF-STEM; Figure 1b–d) confirmed that most of Pt was presented as isolated atoms on the support, with negligible cluster formation. Electrocatalytically, the C_60_-based supported catalyst, Pt/C_60_-2/KB (KB = Ketjen Black), exhibited outstanding activity, requiring only 25 mV overpotential at 10 mA cm^−2^ in 1 M KOH (Figure 1e), outperforming 20 wt% Pt/C (39 mV) and demonstrating superior mass activity. The significantly lower activity of a physically mixed control sample confirmed the essential role of the direct integration onto the C_60_ support. Specifically, Pt single atoms (Pt SAs) can bind with C60 via an η^2^-π bonding mode. This not only prevents the formation of aggregated particles but also facilitates electron transfer between the metal and C_60_, thereby lowering the adsorption energy barrier between water and intermediates. Furthermore, the C_60_ support ensured remarkable stability: negligible degradation after 3000 CV cycles (Figure 1f) and stable operation over 100 h at 10 mA cm^−2^ (Figure 1g), drastically surpassing commercial Pt/C.

The anchoring capability of the support material and its coordination mode with metal atoms critically govern the stability and catalytic performance of SACs. This underscores that the exceptional functionality of such catalysts is fundamentally dependent on the support [45,46,47]. Beyond carbon-based materials, transition metal compounds including oxides, sulfides, and MXenes have emerged as prominent supports for Pt single atoms. Zhou et al. developed a Ni_3_S_2_-supported Pt catalyst achieving a mass activity of 7.6 A mg^−1^—27 times higher than commercial Pt/C in HER [48]. Separately, Huang et al. anchored Pt onto carbon-modified RuO_2_ nanorods (denoted Pt/RuO_2_@C), where RuO_2_ acts as an electronic mediator to facilitate Pt deposition [49]. This catalyst exhibited an ultra-low overpotential of 18 mV at 10 mA cm^−2^, outperforming Pt/C (47.1 mV) by 29 mV. TiO_2_ is also one of the most widely used supports for single-atom decoration. Zhou et al. obtained a single-atom Pt catalyst by depositing Pt on the titanium dioxide (TiO_2_) layer by direct current (DC) sputtering on graphene. TiO_2_ obtained a good HER effect by anchoring high-density Pt with a high specific surface area; the single-atom Pt on TiO_2_ showed an overpotential of only 30 mV. At the same time, TiO_2_ has a wider range of applications in the field of photocatalytic hydrogen evolution [50]. Zhang et al. further immobilized Pt atoms on bimetallic MXene nanosheets synthesized via electrochemical exfoliation [51]. Owing to the strong covalent anchoring by the MXene support, the catalyst demonstrated a 40-fold enhancement in mass activity relative to commercial Pt/C.

### 2.2. Coordination Environment

Altering the coordination number of metal atoms entails a change in their oxidation state, thereby enhancing hydrogenation activity [52,53,54]. Ren et al. demonstrated precise coordination environment tuning in a Fe_2_O_3_-supported Pt SAC [55]. Their approach involved ethylenediamine-assisted chelation of Pt cations, followed by rapid thermal treatment (RTT) under an inert atmosphere to remove ligands. Their extended X-ray absorption fine structure (EXAFS) results suggest that a continuous decrease in the R-space of 1.6–1.8 Å with increasing temperatures, reflecting a decrease in Pt-O (or Pt-N) coordination number, thereby increasing the HER activity to unprecedented levels while maintaining chemoselectivity. This contrasts with conventional co-precipitation (CP) methods, where Pt atoms embedded within the Fe_2_O_3_ matrix exhibit limited accessibility, and reductive activation often induces aggregation—necessitating ultra-low Pt loadings (~0.1 wt%) for dispersion stability.

Graphene-based supports also enable dynamic coordination modulation. Yin et al. engineered distinct Pt coordination environments on graphdiyne (GDY) through thermal annealing [56]. Pt-GDY1 (synthesized via K_2_PtCl_4_ reaction at 273 K) featured five-coordinate C_1_–Pt–Cl_4_ species, while annealing at 473 K transformed it into Pt-GDY2 with a four-coordinate C_2_–Pt–Cl_2_ configuration. This GDY-supported coordination restructuring elevated HER activity dramatically: Pt-GDY2 exhibited 3.3-fold and 26.9-fold higher current densities than Pt-GDY1 and Pt/C, respectively, at −0.1 V overpotential. Density-of-states analysis revealed the enhanced performance originated from high Pt 5d-orbital vacancy and near-zero hydrogen adsorption energy (ΔG_H*_ ≈ 0).

Nitrogen-doped carbon architectures further exemplify coordination engineering. Han et al. constructed Pt-N_2_C_2_ sites on customized porous carbon nanofibers (Pt-SA/pCNFs), achieving exceptional structural stability and HER activity [57]. The Pt-SA/pCNF electrode delivered 500 mA cm^−2^ at merely 64 mV overpotential without binders. Similarly, Lee et al. immobilized Pt atoms on NO_2_-functionalized N-doped carbon nanosheets, forming atomically dispersed NO_2_–Pt–Cl_2_ sites [58]. This catalyst outperformed Pt/C with lower overpotential (25 mV at −10 mA cm^−2^), higher specific activity (1.35 A mgPt^−1^), and bigger TOF values (around 3.10 H_2_ s^−1^ per Pt atom).

### 2.3. Synergistic Effect

Despite the inherent tendency of Pt atoms to coalesce into clusters due to high surface energy—a process traditionally believed to reduce catalytic efficiency and metal utilization—recent studies reveal that strategic integration of single atoms with clusters can generate synergistic enhancements [59,60,61]. This suggests that rationally designed Pt single-atom/cluster hybrids may optimize Pt utilization efficiency [62]. Sun et al. engineered such a system by co-anchoring Pt single atoms (Pt_1_) and sub-nanometer clusters (Pt_n_) on multilayer MXene, denoted Pt_1_ + Pt_n_/MXene [63]. The catalyst exhibited a low overpotential of 61.3 mV at 10 mA cm^−2^ and a Tafel slope of 32.5 mV dec^−1^. Crucially, the synergistic interplay between Pt_1_ and Pt_n_ sites modulated the electronic structure of Pt, enhancing H* adsorption and optimizing the hydrogen evolution energy barrier. This resulted in HER performance comparable to that of commercial Pt/C despite lower Pt loading.

**Figure 2:**
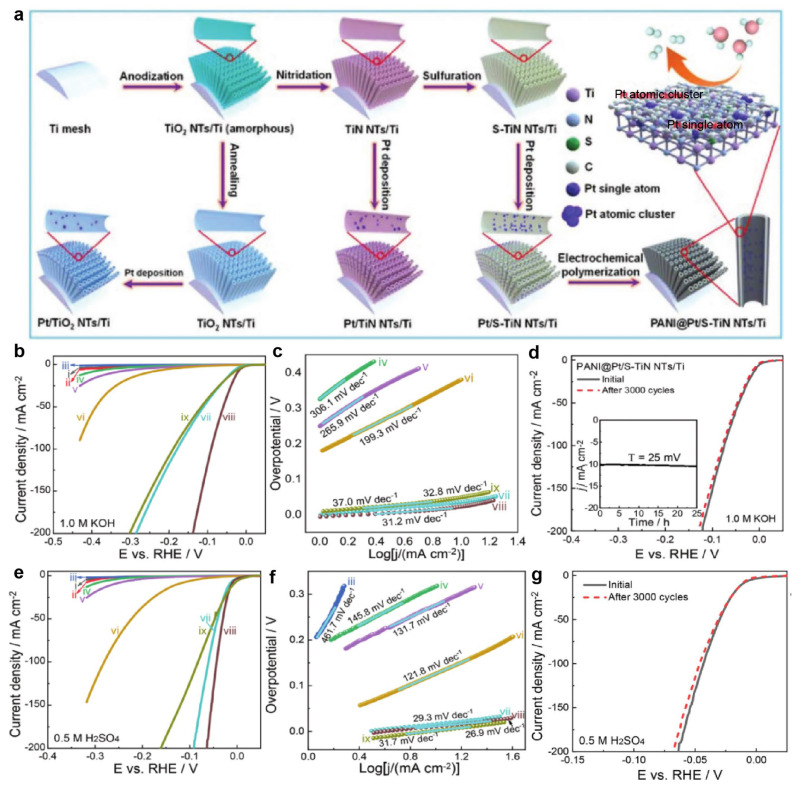
Synthesis and HER performance of Pt/S-TiN NTs/Ti [64]. (**a**) Schematic illustration of the construction of Pt/TiO_2_ NTs/Ti, Pt/TiN NTs/Ti, Pt/S-TiN NTs/Ti, and PANI@Pt/S-TiN NTs/Ti after each step of reactions; (**b**) polarization curves of (i) bare Ti mesh, (ii) S-Ti mesh, (iii) TiN NTs/Ti, (iv) S-TiN NTs/Ti, (v) Pt/TiO_2_ NTs/Ti, (vi) Pt/TiN NTs/Ti, (vii) Pt/S-TiN NTs/Ti, (viii) PANI@Pt/S-TiN NTs/Ti, and (ix) Pt/C/CC recorded in 1.0 M KOH; (**c**) the corresponding Tafel plots recorded in 1.0 M KOH; (**d**) polarization curves of the PANI@Pt/S-TiN NTs/Ti before and after 3000 CV cycles in 1.0 M KOH; (**e**,**f**) polarization curves and Tafel plots for (i) bare Ti mesh, (ii) S-Ti mesh, (iii) TiN NTs/Ti, (iv) S-TiN NTs/Ti, (v) Pt/TiO_2_ NTs/Ti, (vi) Pt/TiN NTs/Ti, (vii) Pt/S-TiN NTs/Ti, (viii) PANI@Pt/S-TiN NTs/Ti, and (ix) Pt/C/CC in 0.5 M H_2_SO_4_ solution at 2 mV s^−1^; (**g**) polarization curves of the PANI@Pt/S-TiN NTs/Ti before and after 3000 CV cycles in 0.5 m H_2_SO_4_ solution.

Extending this concept, Yan et al. developed PANI@Pt/S-TiN NTs/Ti by depositing Pt single atoms and nanoclusters onto sulfur-modified TiN nanotube arrays (Figure 2a) [64]. The deliberate coexistence of Pt_SA_ and Pt_AC_ sites enabled exceptional bifunctional activity: polarization curves (Figure 2b,e) and Tafel plots (Figure 2c,f) demonstrated superior HER performance in both 0.5 M H_2_SO_4_ and 1.0 M KOH electrolytes. DFT analysis quantified the synergy: Pt_SA_/TiN (|ΔG_H*_| = 0.50 eV), Pt_AC_/TiN (Pt1: 0.51 eV; Pt2: 0.91 eV), and Pt_SA_ + Pt_AC_/TiN sites exhibited optimized hydrogen adsorption energetics. The synergistic configuration significantly outperformed catalysts containing exclusively single atoms or clusters, confirming that electronic coupling between adjacent Pt_SA_ and Pt_AC_ sites reduces reaction energy barriers. After 3000 cycles of CV scanning, Figure 2d,g indicate that PANI@Pt/S-TiN NTs/Ti have excellent stability during long-term HER processes, whether under acidic or alkaline conditions.

## 3. Co-Based Catalysts

Co SACs demonstrate significant promise for HER [65,66], particularly in alkaline or neutral electrolytes where their structural stability surpasses that of nanoparticle analogues. Moreover, compared to benchmark Pt-based catalysts—the prevailing choice for HER—Co SACs offer substantial cost advantages due to cobalt’s higher natural abundance [67,68]. Remarkably, recent studies report HER activities of optimized Co SACs approaching or even exceeding those of commercial Pt/C catalysts [69,70,71].

### 3.1. Supports

MoO_3_ exhibits high specific surface area, excellent thermal stability, and favorable electronic conductivity, making it an ideal support for anchoring metal single atoms. Kim et al. synthesized Co single atoms on MoO_3_ (Co SA/MoO_3_) via an amorphous-to-crystalline phase transition strategy [72]. Briefly, anoxic amorphous MoO_3_ was deposited on nickel foam by thermal evaporation under argon, followed by immersion in 0.05 M CoCl_2_/ethanol solution to adsorb Co^2+^ cations. The unsaturated sites in amorphous MoO_3_ effectively trapped and anchored isolated Co ions. Subsequent annealing at 420 °C induced crystallization of MoO_3_ and oxidation of Co, dispersing Co atoms within the MoO_3_ lattice via chemical bonding. This catalyst demonstrated superior HER performance in both acidic and alkaline media. In acidic electrolyte, the overpotential of Co SA/MoO_3_ was only 46% and 35% of that for Co cluster/MoO_3_ and bare MoO_3_, respectively. Similarly, in alkaline medium, it achieved 53% and 47% of the overpotentials required by Co cluster/MoO_3_ and bare MoO_3_. The intrinsic activity was evaluated by turnover frequency (TOF). At 87 mV, the TOF value of Co single-atom MoO_3_ (Co SA MoO_3_) is 0.54 s^−1^, which is more than three times that of Co cluster MoO_3_ (0.17 s^−1^) and bare MoO_3_ (0.13 s^−1^). Notably, Co SA/MoO_3_ outperformed all reported Co single-atom and Mo-based HER electrocatalysts at the time.

Nitrogen-containing carbon materials act as effective single-atom catalyst supports. Their high surface area, ordered/hierarchical porous structures, and optimized preparation conditions (e.g., carbonization temperature, metal loading) enhance catalytic efficiency by maximizing active site exposure, enriching defects, and facilitating reactant/product mass transfer. Nitrogen-assembled carbon (NAC) with ordered porosity was designed by Yu et al. as a support for single-atom catalysts [65]. Among various metal-NAC systems (Fe, Co, Cu, Ru, Ni), Co-NAC exhibited optimal alkaline HER activity, characterized by minimal overpotential and rapid current increase. The high surface area of NAC maximized active site exposure and minimized mass transfer limitations, with Co-100-NAC-800 showing peak performance through optimized Co loading and carbonization temperature (800 °C). Pan et al. further developed N-doped carbon supports via a doping–adsorption–pyrolysis approach, achieving atomic Co dispersion (Co SAs/CN) with a pore volume of 0.36 cm^3^ g^−1^ and an average pore size of 1.67 nm [73]. This hierarchical porous structure enriched defect sites, exposed abundant active centers, and facilitated reactant/product mass transfer, collectively enhancing catalytic efficiency.

### 3.2. Coordination Environment

**Figure 3:**
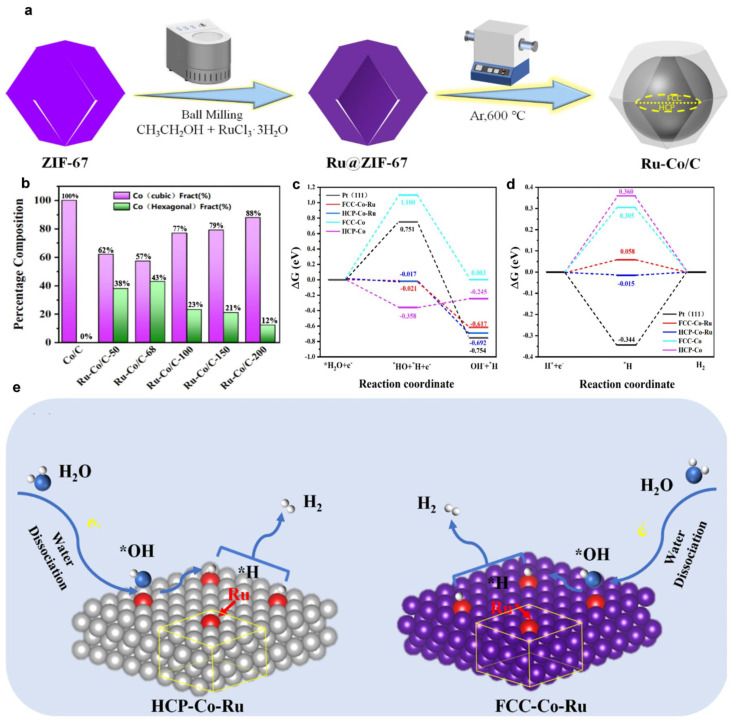
Synthesis method of Ru-Co/C-n and hydrogen evolution mechanism of its internal structure [77]. (**a**) Schematic illustration of the synthesis of Ru-Co/C-n; (**b**) content of FCC and HCP cobalt in samples; (**c**,**d**) free energy diagram for HER on Pt (111), pure FCC-Co and HCP-Co, Ru-doped HCP-Co and FCC-Co; (**e**) mechanism diagram of the HER reaction on the surfaces of Ru-doped HCP-Co and FCC-Co.

The metal structure exhibits inherent selectivity and sensitivity. Modifying the coordination environment of supported metal atoms through crystal structure engineering represents an emerging strategy to simultaneously enhance catalytic activity and active site density in HER catalysis [74,75,76]. Zhang et al. demonstrated that ruthenium doping induces lattice tensile strain in Ru-Co alloys [77], promoting mixed hexagonal close-packed (HCP) and face-centered cubic (FCC) phases for enhanced catalytic performance. The synthesis involved solvothermal preparation of ZIF-67, followed by high-energy ball milling with RuCl_3_·3H_2_O in ethanol to infiltrate ruthenium, yielding Ru@ZIF-67. Subsequent carbonization at 600 °C under argon produced duplex cobalt crystals (Figure 3a). Analysis revealed that HCP phase content initially increased and then decreased with rising Ru concentration (Figure 3b), attributable to Ru-induced thermal destabilization of cobalt and residual stress from mechanical processing. This non-equilibrium state regulation by Ru increased active site exposure. DFT calculations (Figure 3c,d) showed Ru-doped HCP-Co and FCC-Co surfaces follow Volmer–Tafel HER pathways (Figure 3e). Compared to Pt(111), undoped cobalt phases, FCC-Co-Ru and HCP-Co-Ru, exhibited superior reaction energetics: lower water dissociation barriers (−0.021 and −0.017 eV), efficient OH desorption (−0.617 and −0.690 eV), and near-optimal hydrogen adsorption energies (−0.015 eV and 0.058 eV).

Non-metallic coordination plays a pivotal role in constructing cobalt-based single-atom catalysts, as it stabilizes atomically dispersed cobalt sites, facilitates the formation of dynamic active structures, creates low-coordination centers, and enhances the catalysts’ HER kinetic performance. Cao et al. anchored cobalt atoms on phosphocarbonitride (PCN) to fabricate Co_1_/PCN [78], where operando XAFS confirmed dynamic active site structures. The catalyst achieved turnover frequencies of 0.22 s^−1^ (50 mV) and 5.98 s^−1^ (100 mV), significantly surpassing commercial Pt/C while maintaining high stability. HAADF-STEM and elemental analysis verified atomic cobalt dispersion stabilized by Co-N/P coordination. Linear sweep voltammetry revealed a Tafel slope of 52 mV dec^−1^, approaching that of Pt/C (38 mV dec^−1^), indicating favorable kinetics. In a parallel approach, Song et al. engineered asymmetric Co-N_3_ sites using CO (from NaHCO_3_ pyrolysis) to etch graphene [79], creating defect-adjacent low-coordination centers. This yielded exceptional HER metrics: 78 mV overpotential at 10 mA cm^−2^, 45.2 mV dec^−1^ Tafel slope, and 1.67 s^−1^ TOF at 100 mV, establishing it among the most active cobalt-based HER catalysts.

## 4. Ru-Based Catalysts

Despite sharing platinum’s limitations of scarcity and high cost in HER catalysis, ruthenium—as a transition metal—maintains exceptional hydrogen evolution activity at high current densities [80,81,82]. Notably, ruthenium exhibits strong synergistic interactions with other atoms, serving as a viable strategy for optimizing Ru-based single-atom catalysts [83,84,85].

### 4.1. Synergistic Effect

**Figure 4:**
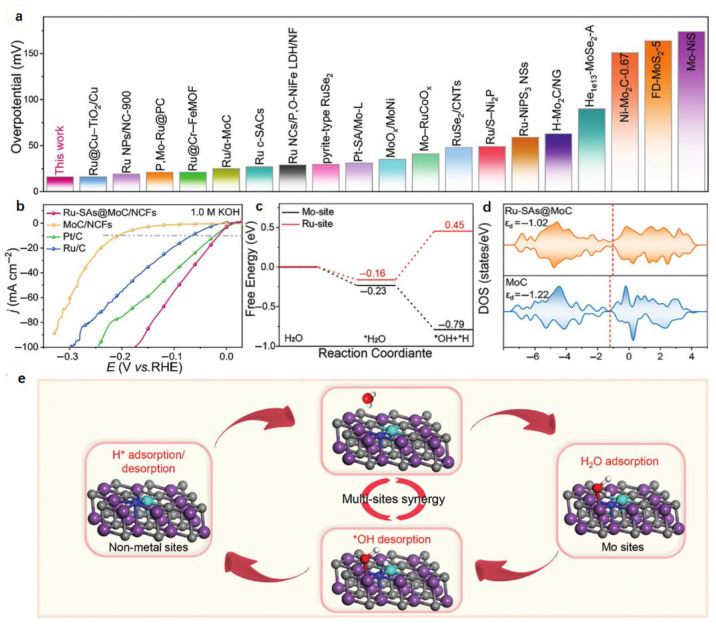
Mechanism studies and HER performance evaluation of Ru-SAs@MoC/NCFs for alkaline HER [89]. (**a**) Activity comparison of Ru-SAs@MoC/NCFs with reported Mo-based and Ru-based catalysts; (**b**) LSV polarization curves; (**c**) Gibbs free energy diagrams of HER paths on Mo-site and Ru-site; (**d**) DOSs of Ru-SAs@MoC and MoC; (**e**) the proposed catalytic mechanism of Ru-SAs@MoC in alkaline HER (the purple, grey, blue, and cyan colors represent Mo, C, N, and Ru, respectively).

Heterostructures exhibit significant potential in catalytic energy conversion due to synergistic effects between components that modulate electronic structures and interfacial charge distribution to enhance surface catalysis [86,87,88]. Yin et al. anchored Ru atoms onto nano-MoC/carbon carriers (MoC/NCFs), creating Ru-SAs@MoC/NCFs with exceptional alkaline HER performance [89]. Ru-SAs@MoC/NCFs exhibited superior catalytic activity relative to previously reported Mo-based and Ru-based HER electrocatalysts (Figure 4a). In 1.0 M KOH (Figure 4b), this catalyst demonstrated a minimal overpotential of 16 mV at 10 mA cm^−2^, substantially lower than that of pristine MoC/NCFs (207 mV) and outperforming commercial Pt/C (23 mV) and Ru/C (67 mV). DFT calculations (Figure 4c) revealed favorable water adsorption Gibbs free energies at Mo (−0.23 eV) and Ru sites (−0.16 eV), indicating preferential water dissociation/adsorption at Mo sites. DOS (Figure 4d) shows that the introduction of a single Ru atom has an electron modulation effect on Mo, which shifts the center of the d band of Mo upward and enhances the water adsorption effect of the Mo site. Meanwhile, negatively charged N sites showed strong H* adsorption capacity. The synergistic interplay among Ru, Mo, and N sites (Figure 4e)—achieved by triggering the activity of three types of sites through Ru doping induction at the atomic level—constitutes the fundamental mechanism for the high catalytic efficiency in alkaline media.

Bimetallic single-atom catalysts featuring Ru paired with other metals demonstrate exceptional pH-universal stability and hydrogen evolution rates. Recent work reports a pyrolytically synthesized Ru-Bi single-atom catalyst on graphene oxide (RuBi SAA/Bi@OG) as an efficient alkaline HER electrocatalyst [90]. At 150 mV overpotential, it achieves a mass activity 72.2-fold higher than commercial Pt/C (65,000 mA mg^−1^) and exhibits optimal hydrogen adsorption free energy (−0.37 eV) at Ru sites due to bimetallic synergy.

Notably, strong Ni-Ru synergy has been demonstrated through controlled bimetallic single-atom engineering on MoS_2_ (Ru/Ni-MoS_2_) [91]. The Ru sites facilitate the chemisorption of OH^−^, while the Ni sites promote the adsorption of H; these two effects synergistically enhance both the hydrolysis and HER processes. Ru/Ni-MoS_2_ achieves an ultra-low overpotential of 32 mV at 10 mA cm^−2^, surpassing both Ni-MoS_2_ (120 mV) and Ru-MoS_2_ (88 mV), and outperforming commercial 5% Pt/C (54 mV).

### 4.2. Coordination Environment

**Figure 5:**
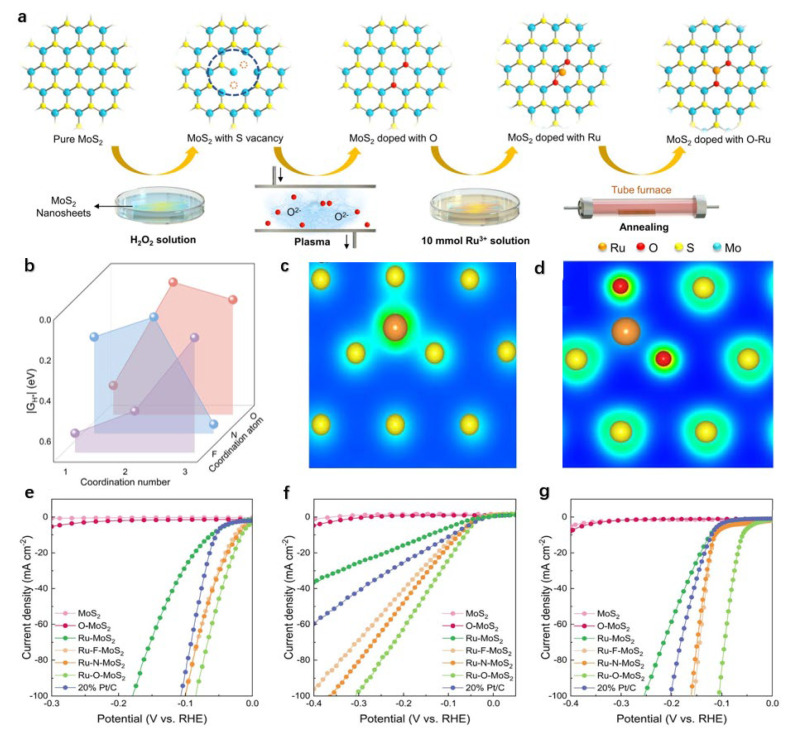
Synthesis, DFT calculations, and HER performance of Ru–X–MoS_2_ [94]. (**a**) Schematic representation of the synthesis process for Ru–O–MoS_2_; (**b**) ΔG_H*_ of Ru–X–MoS_2_ with different numbers of non-metal metal (N, O, and F) coordination; electron concentration distribution of (**c**) S_3_–Ru and (**d**) O_2_–Ru–S_1_; HER polarization curves in (**e**) acidic, (**f**) neutral, and (**g**) alkaline electrolytes.

Modulating the coordination environment of Ru atoms through heteroatom doping represents a key strategy to enhance the HER performance of Ru-based catalysts by accelerating water dissociation kinetics and optimizing hydrogen adsorption barriers [92,93]. Guo et al. demonstrated this approach by introducing non-metallic heteroatoms (O, N, F) to tailor Ru coordination in MoS_2_ [94]. The synthesis of Ru-O-MoS_2_ (Figure 5a) proceeds through sequential steps: hydrogen peroxide treatment creates sulfur vacancies, O_2_ plasma generates oxygenated functional groups (O-MoS_2_), wet impregnation anchors Ru atoms, and final low-temperature reduction under inert atmosphere yields the catalyst. Density functional theory calculations reveal optimal coordination numbers of 3, 2, and 2 for F, N, and O respectively, with corresponding hydrogen adsorption free energies (ΔG_H*_) of 0.132, 0.124, and 0.046 eV (Figure 5b). The Ru-O configuration exhibits the most favorable thermodynamics for hydrogen evolution. Furthermore, while S atoms form a symmetric S-Ru_3_ coordination (Figure 5c), other heteroatoms disrupt this symmetry and concentrate electrons near S sites (Figure 5d), effectively reducing the water dissociation barrier. These findings establish the O_2_-Ru-S_1_ configuration as the optimal coordination structure.

Electrochemical characterization via linear sweep voltammetry (Figure 5e–g) confirms that Ru-O-MoS_2_ outperforms Ru-MoS_2_ significantly across all pH conditions. Specifically, at a current density of 10 mA cm^−2^, it exhibits overpotentials that are 42 mV lower in acidic media, 62 mV lower in neutral media, and 73 mV lower in alkaline media. Additionally, its mass activity and TOF are substantially higher than that of commercial Pt/C, thus unequivocally validating the critical role of oxygen modulation.

Remarkably, heteroatom coordination can generate novel active sites. Wu et al. discovered that in Ru-NPC catalysts—synthesized through high-temperature pyrolysis (900 °C, Ar) of glucose, dicyandiamide, Ru^3+^ salt, and triphenylphosphine on N,P-doped graphene—the phosphorus sites exhibit exceptional activity [95]. Comparative analysis of four configurations revealed Ru-NPC’s P-site possesses a near-ideal |ΔG_H*_| closer to zero than platinum. This catalyst demonstrates outstanding HER activity in 0.5 M H_2_SO_4_ with a Tafel slope of 59.4 mV dec^−1^ (only 19 mV dec^−1^ above Pt/C), along with remarkable stability evidenced by negligible overpotential drift during 20 h of operation and overlapping LSV curves after 3000 cycles.

Advanced characterization elucidates the atomic-scale structure: FT-EXAFS and WT-EXAFS confirm atomic Ru dispersion with Ru-N bonding, while EXAFS fitting (coordination number 3.8) and XANES analysis collectively establish a unique Ru-N_4_-P_1_ configuration. In this ternary microstructure, N atoms occupy Ru’s primary coordination sphere while P resides at the secondary P_1_ site. In situ synchrotron radiation IR spectroscopy further reveals that time-invariant absorption peaks correspond to P-Hads formation, confirming the P atom functions as a highly active non-metallic site enabled by the Ru-N_4_-P coordination.

Beyond non-metallic dopants, secondary metal sites provide another effective coordination engineering pathway. Rong et al. developed a Ru/Co dual-site catalyst on N-doped carbon (Ru/Co–N–C) where atomically dispersed Co-N_4_ sites modulate Ru’s electronic structure [96]. Extended X-ray absorption fine structure (EXAFS) measurements showed that Ru/Co–N–C had a main peak at about 1.48 Å, and no main peaks were observed at 2.3 Å, confirming the formation of single-atom Ru and that no Ru nanoparticles existed in the sample. First-principles calculations indicate this configuration enhances Ru-O covalency while increasing electron density around Ru centers, thereby weakening hydrogen intermediate binding. This electronic synergy yields exceptional pH-universal performance, achieving ultra-low overpotentials of 13 mV (0.5 M H_2_SO_4_) and 23 mV (1 M KOH) at 10 mA cm^−2^.

## 5. Ni-Based Catalysts

Ni SACs demonstrate unique catalytic advantages in HER due to their atomically dispersed active sites and tunable coordination environments (e.g., Ni-N_x_, Ni-S_x_) [97,98,99]. These catalysts maximize active site utilization and optimize electronic structures through mechanisms such as d-band center modulation. This effectively balances the adsorption/desorption energy barriers of reaction intermediates, enabling efficient hydrogen generation activity [100,101].

### 5.1. Supports

Unlike precious metals (e.g., Pt, Ir, Au) with highly delocalized d-electrons, non-precious nickel exhibits partially filled d-orbitals featuring reduced electron delocalization. This inherent electronic structure weakens interactions between non-noble metal single-atom catalysts (NNMSACs) and support matrices, making the identification of suitable Ni-anchoring supports a critical challenge [102,103]. Qiu et al. addressed this by anchoring Ni single atoms onto nanoporous graphene (np-G) via chemical vapor deposition (CVD), followed by chemical etching of excess Ni in 2.0 M HCl [104]. High-angle annular dark-field scanning transmission electron microscopy (HAADF-STEM) confirmed the atomic dispersion of Ni at carbon lattice sites. The resulting catalyst demonstrated exceptional HER performance in 0.5 M H_2_SO_4_, exhibiting a Tafel slope of 45 mV·dec^−1^—marginally higher than that of Pt/C (30 mV·dec^−1^). Remarkably, the cathode current showed negligible decay after 1000 cycles at 150 mV overpotential. This stability arises from two synergistic mechanisms: (1) robust Ni–C covalent bonding via charge transfer, and (2) stabilization through orbital overlap and partial density-of-states (pDOS) interactions between Ni and three adjacent carbon atoms.

Pyrolytically derived N-doped porous carbon from metal–organic frameworks (MOFs) offers high conductivity and abundant defect sites, serving as an effective support. Fan et al. pyrolyzed Ni-MOF at 700 °C under N_2_ to obtain Ni@C, then removed excess Ni with HCl before electrochemical activation to generate atomic Ni sites (A-Ni-C) [105]. Activated A-Ni-C achieved a Tafel slope of 41 mV·dec^−1^ in acidic media—comparable to that of Pt/C and substantially lower than that of HCl-treated Ni@C (194 mV·dec^−1^). This enhancement stems from strong chemical/electronic coupling between graphitized carbon and Ni single atoms induced by electrochemical activation. Electrochemical impedance spectroscopy corroborated this mechanism, revealing charge transfer resistance for A-Ni-C similar to that of Pt/C but significantly lower than that of HCl-Ni@C, indicating accelerated electron transfer at active sites.

**Figure 6:**
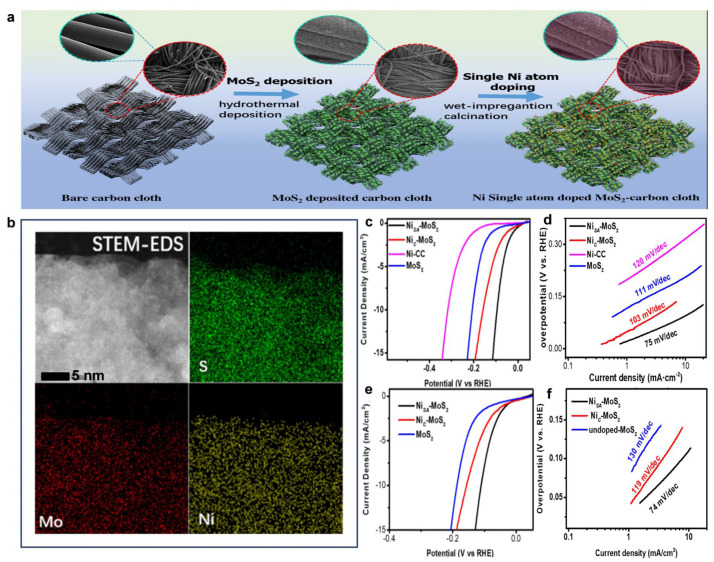
The synthetic scheme, EDS-mapping, and HER performance of Ni_SA_-MoS_2_/CC [106]. (**a**) Schematic procedure for the preparation of Ni_SA_-MoS_2_ and NiC-MoS_2_. The green color of the cartoon represent the MoS_2_ deposited catalyst, and the orange dots correspond to the Ni single-atom decorating of the MoS_2_. The circles in the top figure correspond to the SEM images of the sample at each stage of the preparation. (**b**) EDS-mapping of Ni_SA_-MoS_2_/CC. (**c**) Polarization curves of Ni_SA_-MoS_2_/CC, NiC-MoS_2_/CC, Ni/CC, and MoS_2_/CC in 1 M KOH solution with a scan rate of 5 mV s^−1^. (**d**) Tafel plots obtained from the polarization curves in (**c**). (**e**) Polarization curves of Ni_SA_-MoS_2_, NiC-MoS_2_, and bare MoS_2_ in 0.5 M H_2_SO_4_ solution with a scan rate of 5 mV s^−1^. (**f**) Tafel plots obtained from the polarization curves in (**e**).

As a highly promising Pt-like HER catalyst support, MoS_2_ can stabilize atomically dispersed Ni through its structure and work synergistically with Ni single atoms to alter the rate-determining step of the reaction. Wang et al. engineered atomic Ni sites on MoS_2_ nanosheets (Ni_SA_-MoS_2_/CC) via hydrothermal growth on carbon cloth followed by NiCl_2_ impregnation and H_2_/Ar reduction (Figure 6a) [106]. STEM-EDS elemental mapping confirmed atomic Ni dispersion without clustering (Figure 6b). In 1 M KOH, Ni_SA_-MoS_2_/CC exhibited minimal overpotential (Figure 6c) and a Tafel slope of 75 mV·dec^−1^, outperforming pristine MoS_2_/CC (111 mV·dec^−1^) and a Ni-cluster-modified carbon cloth (120 mV·dec^−1^) (Figure 6d). This performance signifies a shift in the rate-determining step to H_ads_ + H_ads_ → H_2_ (Tafel mechanism), where Ni single atoms accelerate hydrogen recombination. Identical enhancement trends observed in 0.5 M H_2_SO_4_ confirm the pH-universal efficacy of atomic Ni decoration, which surpasses that of Ni clusters (Figure 6e,f). It is worth mentioning that after 2000 CV cycles, the EDS results showed that the loss of Ni single atoms was much smaller than that of Ni clusters, and the stability of Ni single atoms was attributed to the strengthening of Ni-S bonds.

### 5.2. Synergistic Effect

**Figure 7:**
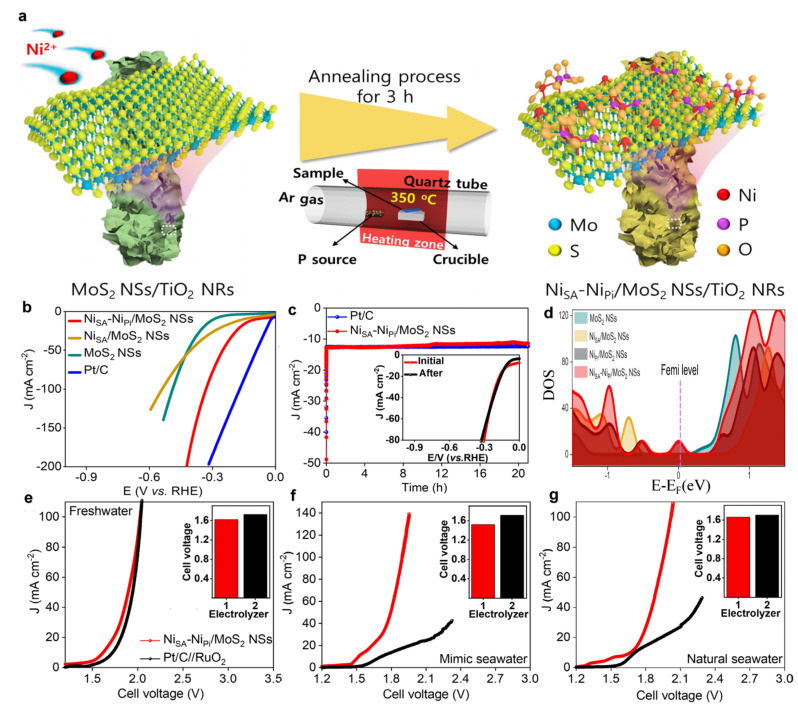
The synthetic scheme and HER performance of Ni_SA_-Ni_Pi_/MoS_2_ NSs [110]. (**a**) Schematic sketch for the fabrication of the Ni_SA_-Ni_Pi_/MoS_2_ NSs material synthesized on 1D-TiO_2_/CC substrate; (**b**) iR-corrected LSV responses of the synthesized catalysts and commercial Pt/C toward HER at a scan rate of 10 mV s^−1^ in freshwater with 1.0 M KOH condition; (**c**) amperometry for HER stability of the Ni_SA_-Ni_Pi_/MoS_2_ NSs catalyst (inset: LSV measurement of the Ni_SA_-Ni_Pi_/MoS_2_ NSs catalyst before and after HER stability test); (**d**) DOS calculation for the material models; LSV measurement of the Ni_SA_-Ni_Pi_/MoS_2_ NSs_(+,−)_ and Pt/C_(−)_//RuO_2(+)_-based electrolyzers at 10 mV s^−1^ in (**e**) freshwater, (**f**) mimicked seawater, and (**g**) natural seawater under the 1.0 M KOH condition.

Synergistic interactions between Ni single atoms and adjacent clusters or heterometals enhance adaptability to complex reaction pathways [107,108,109]. Kim et al. synthesized MoS_2_ nanosheet-coated 1D-TiO_2_ hydrothermally [110], subsequently adding Ni^2+^ and a phosphorus source followed by reduction at 350 °C under Ar for 3 h to obtain Ni_SA_-Ni_Pi_/MoS_2_ NSs—a system co-supporting single Ni atoms (Ni_SA_) and nickel phosphate clusters (Ni_Pi_) (Figure 7a). Linear sweep voltammetry revealed an overpotential of 94 mV at 10 mA·cm^−2^, superior to that of MoS_2_ NSs (291 mV) and Ni_SA_/MoS_2_ NSs (152 mV), though higher than that of Pt/C (19 mV) (Figure 7b). The catalyst exhibited 21 h electrochemical stability with 90.4% current retention, comparable to that of Pt/C (~93.1%) (Figure 7c). DOS (Figure 7d) analyses demonstrated that Ni_SA_-Ni_Pi_ synergy enhances metallicity to facilitate charge transfer while optimizing hydrogen adsorption free energy (ΔG_H_*), collectively boosting HER performance. Notably, Ni_SA_-Ni_Pi_/MoS_2_ NSs showed exceptional efficiency in seawater electrolytes, requiring cell voltages of only 1.52 V (simulated seawater) and 1.66 V (natural seawater) at 10 mA·cm^−2^, outperforming Pt/C_(−)_//RuO_2(+)_ counterparts (1.71 V and 1.70 V, respectively) (Figure 7e–g).

**Figure 8:**
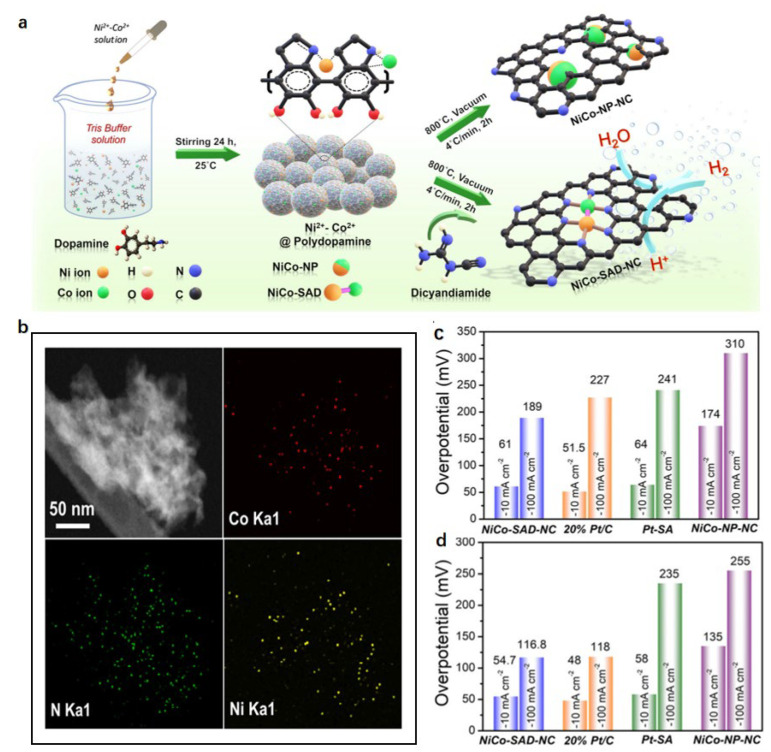
The synthetic scheme, electron microscopy, and HER performance of NiCo-SAD-NC [111]. (**a**) Schematic illustration of the synthetic strategy for the NiCo-SAD-NC and NiCo-NP-NC: (I) trapping metal ions (Ni and Co) into the polydopamine (PDA) via self-polymerization of dopamine; (II) generation of NiCo-NP-NC and NiCo-SAD-NC through calcination treatment upon modulating nitrogen containing precursor (dicyandiamide) amount; (**b**) HAADF-STEM image and corresponding EDS maps of NiCo-SAD-NC showing the uniform dispersion of N (green), Co (red), and Ni (yellow); (**c**,**d**) the overpotentials required to reach −10 and −100 mA cm^−2^ in 1 M KOH and in 0.5 M H_2_SO_4_, respectively.

Constructing heterometallic diatomic sites (single-atom dimers, SADs) creates unique atomic interfaces with bimetallic synergy. Kumar et al. immobilized Ni/Co ions in polydopamine (PDA) via dopamine self-polymerization, then pyrolyzed with dicyandiamide at 800 °C for 2 h to obtain NiCo-SAD-NC (Figure 8a) [111]. According to Figure 8b, the HAADF-STEM and EDS element profiles show that N, Ni, and Co atoms are evenly dispersed in NiCo-SAD-NC rather than aggregating any possible aggregates in the form of NPs. This catalyst demonstrated exceptional HER activity in both 0.5 M H_2_SO_4_ and 1 M KOH. In alkaline media, it required overpotentials of merely 61 mV and 189 mV to achieve −10 and −100 mA·cm^−2^, surpassing 20% Pt/C. In acid, low overpotentials of 54.7 mV (η10) and 116.8 mV (η100) were comparable to those of Pt/C (48 mV and 118 mV) (Figure 8c,d). DFT calculations attribute this to the synergistic Ni-Co interaction: the emergence of Ni-Co bonds with strong electronic coupling at the atomic level constructs the Ni-Co atomic interface, which shifts the center of the d-band towards the Fermi level, accelerating the dissociation kinetics of water.

Heterostructure engineering further amplifies synergistic effects. Feng et al. developed a multi-component electrocatalyst (N–C@CoP/Ni_2_P) featuring N-doped carbon-coated CoP/Ni_2_P heteronanoparticles with atomic Co/Ni sites [112]. The CoP/Ni_2_P heterointerface creates extensive coupling regions that (1) enhance synergistic interactions to improve catalytic activity and (2) enable cooperative reaction pathway sharing between components.

## 6. Other Catalysts

Beyond the previously discussed Pt-, Ru-, Co-, and Ni-based single-atom catalysts, other single-atom catalysts are also promising catalytic materials for HER [113,114,115]. Wang et al. fabricated Fe single atoms anchored on N-doped porous carbon (Fe-N_4_/NPC) through pyrolysis of bimetallic Fe/Zn-phthalocyanine, where volatile Zn species evaporate at elevated temperatures. This catalyst exhibits remarkable HER performance in alkaline electrolyte (1 M KOH), requiring only 202 mV overpotential to reach 10 mA·cm^−2^ while maintaining robust stability [116]. Yang et al. demonstrated that tailored N-coordination can modulate the electronic structure of single-atom Cu catalysts to obtain high HER performance. Their study revealed that Cu_2_N_4_@graphene with p-orbital hybridization achieves optimal HER performance, whereas both higher and lower Cu-N coordination numbers degrade activity. Density functional theory calculations confirm a near-ideal hydrogen adsorption free energy (ΔG_H*_ = –0.09 eV) for this configuration. Projected p-orbital density analysis further indicates that the Cu_2_N_4_@graphene p-band center aligns optimally with hydrogen adsorption energy levels, maximizing adsorption intensity [117]. Besides Fe-based and Cu-based single-atom catalysts, it has been reported that single-atom W catalysts with W-N4 structure deliver excellent catalytic performance for HER, which achieves an ultra-low overpotential of 85 mV with a Tafel slope of 53 mV·dec^−1^ at 10 mA·cm^−2^ [116].

## 7. Conclusions and Outlook

Electrochemical water splitting represents a green pathway for hydrogen production, where SACs have emerged as pivotal materials for accelerating the HER performance. SACs maximize the atomic utilization efficiency, significantly reducing costs. Beyond economic advantages, their unique electronic structure provides excellent catalytic performance for HER. Numerous studies indicate that SACs outperform commercial Pt/C nanocatalysts, demonstrating immense application potential through metal–support interactions, strategic coordination environment engineering, and synergistic effects. The catalyst support forms the critical architectural framework governing the HER performance. The strong interaction between the active metals and supports can stabilize the geometric structure of SACs and regulate their electronic structure. Additionally, precise tailoring of the coordination environments is also an important way to improve HER performance. The regulable coordination environments of SACs include the type of coordination atoms, bond distance, and coordination number. Finally, multi-component synergy of SACs represents the key frontier to further improve HER performance. Strategic effects of single-atom sites/metal clusters, bimetallic single-atom pairs, or single-atom sites/non-metal sites enable SACs to achieve higher catalytic performance for HER.

Even though SACs are shown to exhibit excellent catalytic performance for HER, there are still many challenges for developing SACs. Firstly, since the active sites of SACs are very small, precise structure identification of SACs is very difficult. Advanced characterization technology plays a key role in further revealing the structure–function relationship. Additionally, at present, synthesis of SACs with a well-defined structure faces great challenges, especially for synthesis at a large scale. Thus, exploration of scale-up strategies to obtain well-defined SACs is of critical importance. Finally, the high HER performance of SACs has only been reported in academic research to date; under severe industrial conditions, SACs may deactivate very quickly, which would limit the further applications of SACs.
